# Microfluidic Vaterite Synthesis: Approaching the Nanoscale Particles

**DOI:** 10.3390/nano13233075

**Published:** 2023-12-04

**Authors:** Ivan Reznik, Mikhail A. Baranov, Sergei A. Cherevkov, Petr V. Konarev, Vladimir V. Volkov, Stanislav Moshkalev, Daria B. Trushina

**Affiliations:** 1 International Research and Education Centre for Physics of Nanostructures, ITMO University, 197101 Saint Petersburg, Russia; mbaranov@mail.ru (M.A.B.); s.cherevkov@itmo.ru (S.A.C.); 2 Faculty of Electrical Engineering and Computing, University of Campinas, Campinas 13083-970, Brazil; 3 Federal Scientific Research Centre “Crystallography and Photonics”, Russian Academy of Sciences, 119333 Moscow, Russia; peter_konarev@mail.ru (P.V.K.); volkicras@mail.ru (V.V.V.); 4 Centre for Semiconductor Components and Nanotechnology, University of Campinas, Campinas 13083-870, Brazil; stanisla@unicamp.br; 5 Institute of Molecular Theranostics, Sechenov First Moscow State Medical University, 119435 Moscow, Russia; trushina.d@mail.ru

**Keywords:** CaCO_3_, vaterite, microfluidic synthesis, additive manufacturing, nanoparticles, one-phase synthesis, two-phase synthesis

## Abstract

The challenge of continuous CaCO_3_ particle synthesis is addressed using microfluidic technology. A custom microfluidic chip was used to synthesize CaCO_3_ nanoparticles in vaterite form. Our focus revolved around exploring one-phase and two-phase synthesis methods tailored for the crystallization of these nanoparticles. The combination of scanning electron microscopy, X-ray diffraction, dynamic light scattering, and small-angle scattering allowed for an evaluation of the synthesis efficiency, including the particle size distribution, morphology, and polymorph composition. The results demonstrated the superior performance of the two-phase system when precipitation occurred inside emulsion microreactors, providing improved size control compared with the one-phase approach. We also discussed insights into particle size changes during the transition from one-phase to two-phase synthesis. The ability to obtain CaCO_3_ nanoparticles in the desired polymorph form (∼50 nm in size, 86–99% vaterite phase) with the possibility of scaling up the synthesis will open up opportunities for various industrial applications of the developed two-phase microfluidic method.

## 1. Introduction

In the realm of applications, synthetic calcium carbonate particles (such as vaterite) serve as carriers for active compounds in medical treatments and have been explored as templates for biodegradable polymer capsules in nanomedicine [1]. They find use in personal care products for various purposes, for example as abrasives, adsorbents, anticaking agents, buffers, and dyes. Furthermore, vaterite has been proposed as a coating pigment for inkjet paper, owing to its advantageous properties such as easy and cost-effective preparation, the ability to design particles with defined characteristics, a porous structure, mild conditions for decomposition, non-toxicity, and biocompatibility. The continuous synthesis of submicron calcium carbonate particles has been a subject of intense research due to its wide-ranging applications in various industries, including pharmaceuticals, cosmetics, and materials science [2,3,4,5]. However, the achievement of precise control over the size, morphology, and porosity of these particles has posed a significant challenge. Previous attempts to synthesize submicron calcium carbonate particles relied on various methods, including precipitation, hydrothermal synthesis, chemical vapor deposition, and a biomimetic approach [6,7,8,9]. While these techniques have shown some success in producing small calcium carbonate particles, they often suffer from limitations in terms of particle size distribution, shape control, and scalability [10,11]. Precipitation methods, such as the carbonation route, have been widely used for synthesizing calcium carbonate particles [12]. However, these methods typically result in broad size distributions and irregular morphologies due to the rapid and uncontrolled growth of particles in the solution. This approach faces particular challenges in achieving submicron-sized particles with a uniform size and shape. Hydrothermal synthesis, which involves high-temperature and high-pressure conditions, has been explored to obtain submicron calcium carbonate particles with improved control over the particle size and morphology [7,13]. However, the complexity of the process, along with the requirement for specialized equipment, limits its practicality and scalability. Biomimetic synthesis aims to replicate nature’s sophisticated capability to create intricate and impressive solid structures from dissolved substances, all achieved under gentle conditions like near-neutral pH and low temperatures [9,14,15,16]. While biomimetic synthesis holds immense promise in the controlled synthesis of calcium carbonate particles, its complexity, limited scalability, biocompatibility concerns, and longer synthesis times should be carefully considered and addressed in order to harness its full potential. Despite these previous efforts, the synthesis of submicron vaterite particles with well-defined properties and controlled porosity has remained a challenge. The limitations of existing methods highlight the need for innovative approaches that offer improved control, scalability, and efficiency. In the context of synthesizing submicron calcium carbonate particles, microfluidic technology is studied as an innovative approach for successful application. The proposed solution introduces the emulsion microreactors within an oil medium using a microfluidic chip [17]. This approach leverages the advantages of emulsion systems such as enhanced kinetics and confined reaction environments. The microfluidic chip enables precise control over flow rates, mixing ratios, and reaction times. This novel method overcomes previous limitations, offering an efficient and reliable approach for submicron vaterite particle synthesis. By creating a two-phase system, we achieved better control over the particle size, morphology, and porosity. The results presented in this article provide valuable insights into the changes observed in particle properties when transitioning from a single-phase to a two-phase synthesis method, paving the way for further advancements in submicron particle synthesis and its applications in various industries.

## 2. Materials and Methods

### 2.1. Chemicals

Calcium chloride (Sigma Aldrich, St. Louis, MO, USA), sodium carbonate (Vekton, Saint-Petersburg, Russia), deionized water, ethylene glycole (Ekos 1, Moscow, Russia), castor oil, ethanol, and photopolymer resin (Anicubic, Hong Kong, China). The water for the experiments was purified using a Milli-pore system. All of the chemicals were used without further purification.

### 2.2. Microfluidic Chip Formation

**Figure 1:**
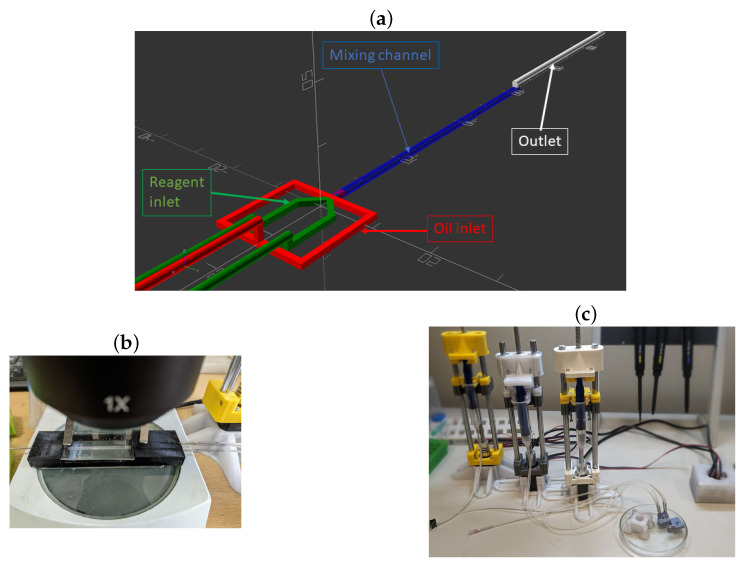
(**a**)—Parametric model of the microfluidic chip with three inputs and one output. The input and channel for oil delivery are highlighted in red. The inputs and channels for precursor delivery into the chip are marked in green. The glass tube, where the formation of microbubbles with the reaction mixture occurs, is marked in blue. (**b**)—Photograph of the assembled chip connected to syringe pumps and placed under the microscope. (**c**)—Photograph of the hardware part of the syringe pump system connected to the microfluidic chip.

The Anicubic Photon Mono 2K printer (Anicubic, Hong Kong, China) and a photopolymer resin were utilized for the fabrication of microfluidic chips using photopolymer 3D printing (MSLA), similar to Ref. [18]. This study utilized a chip with three input channels and one output channel, all connected to the main channel on the chip. Figure 1 shows a 3D model of a cross-section of the microfluidic chip and photos of chips formed using photopolymer 3D printing.

A script was written in OpenScad to generate the microfluidic chip model, allowing for quick parameter adjustments (such as the number of inputs, convergence angle of channels, length, and width). To form physical copies of the chips using additive manufacturing techniques, the chip design was subtracted from the solid block, thus forming the cavities of the microfluidic channels. The main channel on the chip was a glass tube with an inner diameter of 1 mm and an outer diameter of 2 mm, measuring 50 mm in length. It was enclosed between two cover glasses in an aqueous medium, which was used to mitigate image distortion on the curved walls inside the glass channel. One of the input channels was designed for introducing oil, while the other two were intended for introducing water-soluble reagents. The inputs enabled precise dosing and directing of each component into the main channel. In the main channel, the reagents encountered the oil, which led to a chemical reaction of CaCO_3_ crystallization. The output channel was used for collecting the reaction mixture for further analysis. After passing through the main channel, the reaction mixture was poured into an Eppendorf tube with a solvent (water and ethanol). The chip printing parameters for this photopolymer resin were as follows: the thickness of one layer and irradiation time per layer were 50 µm and 2 s, respectively. After printing finalization, the channels of the chip and its surface contained a large amount of unpolymerized resin. To remove it, the chip was placed in a bath filled with isopropanol and exposed to ultrasound for 10 min. After that, the internal channels of the chip were flushed with pure isopropanol using a syringe pump connected to the chip inlet. The syringe pumps were connected to the microfluidic chip using Teflon tubes with inner and outer diameters of 1 and 1.5 mm, and steel adapters with inner and outer diameters of 0.9 and 1.1 mm, respectively. The microfluidic chip connection itself was performed in two stages. In the first stage, one end of the steel adapter was inserted into the Teflon tube and the other end was inserted into the inlet or outlet port of the microfluidic chip. In the second stage, to seal the chip, all of the joints were smeared with several layers of photopolymer resin followed by polymerization of the joints using UV light irradiation with a wavelength of 405 nm for 10–30 s.

### 2.3. Synthesis of CaCO_3_ Particles

To conduct experiments on the microfluidic synthesis of calcium carbonate particles, a programmable hardware system was assembled based on the syringe pump project published by Pierce’s research group and made available in open access [19]. This system includes syringe pumps driven by Nema 17 stepper motors, a microcontroller based on Arduino for motor control, and a Python program that enables the specification of multi-stage and independent operation programs for syringe pumps. For the synthesis of CaCO_3_, solutions of CaCl_2_ and Na_2_CO_3_ in water, as well as mixtures of water and ethylene glycol (in ratios of 1:1, 1:2, and 1:5), were employed. The concentrations of the salts ranged from 0.1 to 1 M. Two types of syntheses were conducted in this study. The first type was a single-phase synthesis without the addition of oil. The total flow rate of the reactants was set at 0.01 mL/s. After passing through the internal volume of the chip, 500 μL of the reaction mixture was poured into an Eppendorf tube containing 1500 μL of water. Subsequently, the synthesized micro-particles were washed using centrifugation (5000 rpm for 5 min), decanted into the supernatant, and the precipitate was redisperced in clean water (this procedure was repeated twice). The second type was a two-phase synthesis with the addition of castor oil to form an emulsion, which provided the nucleation and growth of CaCO_3_ within droplets. The total flow rate of the reactants was 0.001 mL/s, and the oil flow rate was 0.01 mL/s. Ethanol was used to dissolve the oil phase and to precipitate the particles. The resulting particles were washed three times with ethanol (precipitation at 5000 rpm for 5 min).

### 2.4. Characterization Methods

**Figure A1:**
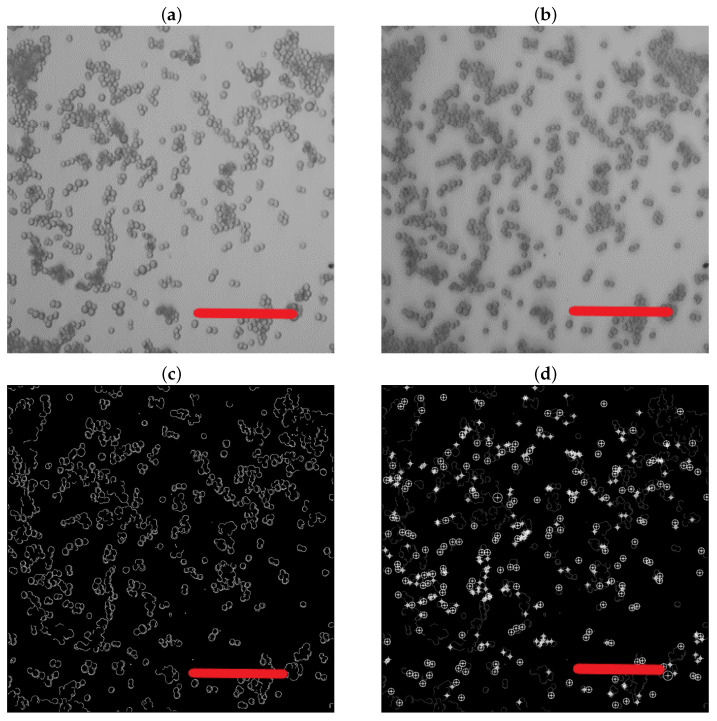
Stages of image processing: the original image taken from the center of the LSM Z-stack (**a**), the image after Z-projection of the entire Z-stack with pixel value averaging based on the minimum value (**b**), the image after applying the Canny edge detection operator (**c**), the image with detected circles after the Hough transform (**d**). Scale bar is 10 μm.

The particle size and morphology analysis of the investigated particles was performed using multiple techniques. Confocal microscopy on the LSM 710 system from Zeiss, Germany provided detailed imaging, while scanning electron microscopy (SEM) enabled high-resolution imaging at 10 kV. A tabletop scanning electron microscope, Hitachi TM4000 (Tokyo, Japan), was used to study the morphology and size of the synthesized particles, as well as a MERLIN (Zeiss) at 20.1 kV with the SE2 detector. Additionally, dynamic light scattering (DLS) measurements were conducted using a Malvern instrument to capture the size distribution information. To gain insight into the phase content transition from vaterite to calcite, complementary methods were employed. Raman spectroscopy was used to examine the molecular structure and composition of the particles, while X-ray structural analysis (XRD) was employed to analyze the crystalline structure and to determine the phase composition. The X-ray diffraction pattern from the nanoparticle powder was collected using a Miniflex 600 X-ray diffractometer with Cu Kα radiation λ = 1.5406 Å, Rigaku, Japan) with a scanning rate of 2${}^{\circ }$/min. We matched the reflections of the experimental diffraction patterns with Inorganic Crystal Structure Database (ICSD) records. The data corresponding to the spatial structure R3a and the trigonal crystal system and the spatial structure P63/mmc and hexagonal symmetry for the calcite and vaterite phases were used, respectively [20,21]. This combination of analytical methods allowed for a comprehensive understanding of both the physical characteristics (size and morphology) and chemical properties (phase composition) that the investigated particles possessed. For a faster and more accurate particle size distribution analysis, a digital image processing routine was implemented. As seen in Figure A1, the use of the Canny and Hough transforms successfully detected circles in the images. This procedure demonstrated a high degree of reproducibility due to the significantly limited number of variables involved in the transformations.

Small-angle X-ray scattering (SAXS) measurements were performed on the automatic small-angle X-ray scattering diffractometer “AMUR-K” (Institute of Crystallography, Moscow, Russia) equipped with a Kratky collimation system and an OD3M one-coordinate position-sensitive gas detector at a fixed wavelength λ = 0.1542 nm (Cu Kα line of a sharp-focus tube, pyrolytic graphite monochromator) [22]. The X-ray beam cross-section was 0.2 × 8 mm. In this geometry, the SAXS measurements provided the range of the momentum transfer 0.11 nm${}^{-1}<$ s < 10.0 nm${}^{-1}$ (where s = 4πsinΘ/λ, 2Θ is the scattering angle and λ is the wavelength). The analyzed powders of the CaCO_3_ samples were measured in a specially designed 1 mm thick cuvette with mylar windows (15 μm thickness); the cuvette was placed in a vacuum chamber. The sample-to-detector distance was 700 mm and the exposure time was 10 min. The experimental SAXS data were normalized to the incident beam intensity, and then a correction for the collimation distortion was made in accordance with the procedure described in Ref. [23]. The scattering from the empty cell was subtracted from the sample scattering intensity using the program PRIMUS [24]. Three programs were used to calculate the size distributions of inhomogeneities in the samples based on the minimization of the total quadratic difference between the experimental and model scattering intensities.

(I) Direct search of particle size distribution was performed by the linear least squares method with Tikhonov regularization of the solution (program GNOM from the ATSAS package) [25,26]. In discrete form, the theoretical scattering intensity is written as follows
  (1) $$I\left(s\right)=\Sigma_{r=R_{min}}^{R_{max}}D_{V}\left(r_{i}\right)\cdot \nu \left(r_{i}\right)\cdot i_{0}\left(s,r_{i}\right)\cdot dr_{i}$$
  where $D_{V}\left(r\right)$ is the desired volume size distribution function defined over size range $r\in [R_{min}:R_{max}]$; $i_{0}\left(s,r\right)$ is the square of the form factor, or the scattering intensity from a particle of a given shape with radius $r_{i}$, and unit volume and density contrast; $\nu (r_{i})$ is the volume of the particle with effective radius $r_{i}$; and $dr_{i}$ is the step on the radius grid. $D_{V}\left(r\right)$ is found as the solution of a system of linear equations with regularization by the first derivative of the distribution.
(II) Direct search of particle size distribution was performed in the form of a histogram (program VOLDIS) [27]. The elements of distribution vector $D_{V}\left(r\right)$ are the search parameters for the minimum of the target function
  (2) $$\min_{x}{\left\|f\left(x,s\right)\right\|}^{2},\mathrm{where}\;f\left(x,s_{i}\right)=\frac{\left(I_{exp}\left(s_{i}\right)-\zeta \cdot I\left(s_{i}\right)\right)\cdot w\left(s_{i}\right)}{\|I_{exp}\left(s_{i}\right)\cdot w\left(s_{i}\right)\|}$$
  where *x* is the vector of current values of the distribution $D_{V}\left(r\right)$, or parameter vector; I(s) is the theoretical intensity according to Formula (1); w(s) is a weighting function that compresses the range of scattering intensities to ratio $I_{max}\left(s\right)/I_{min}\left(s\right)\in \left[5:20\right]$; and ζ is an auxiliary least-squares multiplier that matches the scattering curves before calculating the difference.
(III) A parametric approach based on the use of a superposition of several smooth distributions is given in analytical form (in this paper, Schulz distributions, program MIXTURE from the ATSAS package) [24,28]. The algorithm is also based on the minimization of the function of type (2), where vector *x* is calculated from the sum of several (usually 3–5) smooth analytic functions. The search parameters are the positions and half-widths of such functions and their relative contributions. In this study, we used scattering from a homogeneous sphere, the most commonly used in practice, at the unit contrast and volume as a formulator of inhomogeneities:
  (3) $$i_{0}\left(s,r\right)={\left\{\frac{3\cdot [sin(sr)-sr\cdot cos(sr)]}{{\left(sr\right)}^{3}}\right\}}^{2}$$
  Each of the above algorithms has its advantages and drawbacks, and applying only one of them is unlikely to provide artifact-free solutions. In particular, the distribution curves obtained with the GNOM and VOLDIS programs can show significant oscillations. These oscillations are due to the fact that during the search process, the increase in the scattering contribution from the particles of one radius is compensated by a decrease in the contribution from particles with nearby radii, without changing the shape of the theoretical scattering curve significantly. The MIXTURE algorithm is devoid of this disadvantage, as the resulting distribution curve is the sum of the smooth partial functions. However, the MIXTURE target function is multimodal, which requires setting starting values for the parameters of the partial distributions that are close enough to the global solution. These starting values can be determined through a visual analysis of the solutions obtained with the GNOM and VOLDIS programs, which was conducted in this paper.

## 3. Results

### 3.1. Single-Phase Synthesis in Water and Water-EG Conditions

**Figure 2:**
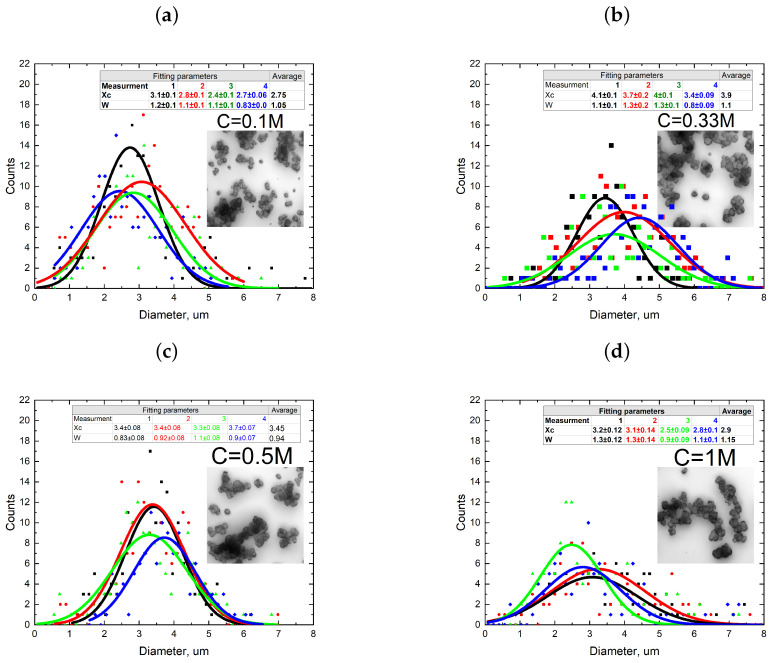
CaCO_3_ size distribution dependency on concentrations of reactants (0.1 M—(**a**), 0.33 M (**b**), 0.5 M (**c**), 1 M (**d**)). Each graph presents four curves corresponding to a separate synthesis with the indicated reactant concentration. The insets show images of particles obtained using a confocal microscope (scale bar is 10 μm) and utilized for size distribution analysis using the methodology described in the Materials and Methods Section 2.4.

At first, we focused on the reproducibility of CaCO_3_ synthesis by varying the concentration of reactants in the range of 0.1 to 1 M. Understanding the reproducibility of the synthesis process and the impact of concentration variations on particle size characteristics is crucial for optimizing production protocols and ensuring consistent product quality. Additionally, the average particle size and size distribution were monitored throughout the experiment. The synthesis procedures were repeated several (3 to 5) times for each process. Figure 2 shows the dependence of particle sizes on the concentration of reagents used in microfluidic synthesis. The insets in Figure 2 show the LSM images of CaCO_3_ particles synthesized by the microfluidic method.

**Figure 3:**
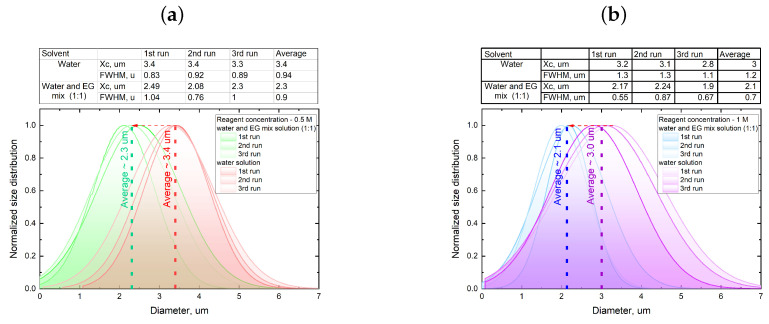
Size distribution comparison between several synthesis iterations of CaCO_3_ microparticles depending on the reagent concentration ((**a**) 0.5 M, (**b**) 1 M) and solution used to prepare the reagents (deionized water for the green and blue shaded curves, and deionized water and EG mix for the red and violet shaded curves). Insets contain the fitting parameters of the experimentally obtained data. Size distribution analysis performed using the methodology described in the Appendix A section on a data set provided by LSM measurements.

In all of the conducted microfluidic one-phase system syntheses, CaCO_3_ particles were predominantly present in vaterite form. As observed from the graphs shown in Figure 2, the single-phase synthesis of CaCO_3_ particles exhibited relatively low reproducibility across all of the concentrations used. In certain cases, particle sizes were observed to vary by a minimum of 0.1 μm and a maximum of 1 μm between different synthesis iterations. Calculating the average particle size based on the sample from the four syntheses revealed that increasing the concentration of reactant salts up to 1 M only slightly reduced the particle size and decreased their size distribution. This finding aligns well with the previously published studies [29]. In summary, the results indicate that the reproducibility of single-phase CaCO_3_ synthesis in the microfluidic system was limited, and particle size variations were significant between different synthesis attempts. However, increasing the concentration of reactant salts contributed to a slight reduction in particle size and improved the reproducibility of results, consistent with earlier findings. One reason for the relatively low reproducibility and micron size of the synthesized particles resulted from the low viscosity of the reactant solutions, which led to more active mixing of salts and rapid particle growth during flow in the microfluidic chip channel. A possible solution to address this issue is to increase salt solutions viscosity by adding ethylene glycol. Based on the data presented in Figure 2, it was decided to use highly concentrated solutions of 0.5 M and 1 M as they yielded the most reproducible results. Another reason to use solutions with a high salt concentration is their reactivity. As the microfluidic chips used here were characterized by a relatively small inner volume, and the reaction time between reagents was limited by the time spent in contact with each other inside the chip, it was reasonable to use more reactive salts at a concentration of 0.5 and 1 M. Consequently, the choice of higher concentrated solutions (0.5 M and 1 M) aimed to achieve a high reaction yield with a more reproducible synthesis result. Such an approach considers the interplay between solution viscosity, mixing behavior, and particle growth dynamics to enhance the reproducibility and size control over the synthesized particles in the microfluidic system. Figure 3 presents a comparison between the size distributions of CaCO_3_ particles synthesized using the reactants dissolved in deionized water and a mixture of deionized water and ethylene glycol at a 1:1 ratio.

The graphs in Figure 3 demonstrate that replacing water with a mixture of water and ethylene glycol as the solvent for reactants resulted in a reduction in the average particle diameter of CaCO_3_ by approximately 30%, regardless of the reactant concentration. This change also led to a decrease in size dispersion, which was particularly noticeable at a reactant concentration of 1 M (size distribution width decreased from 1.2 to 0.7 microns). The addition of ethylene glycol increased the viscosity of the reactant solutions, allowing for better control of the mixing dynamics and promoting better control of particle growth within the microfluidic chip. These findings underscore the significant influence of solvent composition on particle synthesis and size control, and they are in a good agreement with previously published data [29]. However, it should be noted that when transitioning to a more viscous solvent mixture, a slightly greater variation between individual synthesis runs was observed. This could be attributed to increased inertial effects of the volumetric flow rates of reactants encountered at the beginning of the channel, which may influence the efficiency of diffusive mixing at the interface of the two reactants [30].

### 3.2. Two-Phase Synthesis in Water and Water–EG Conditions

**Figure 4:**
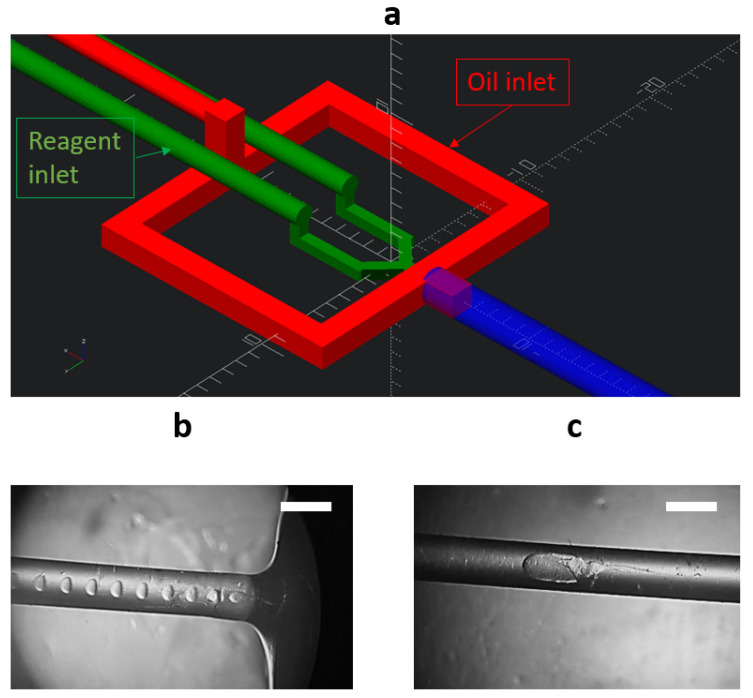
(**a**)—Schematic representation of the internal structure of the microfluidic chip used for the two-phase synthesis of calcium carbonate microspheres. The red indicates the inlet and channels for oil delivery, while the green indicates the inlets and channels for reactant delivery. The reactant channels intersect with each other at a 45-degree angle. The blue represents the glass tube through which the reactant mixture is transported to the chip’s outlet. (**b**,**c**)—Microphotographs of the generated microbubbles when using reactants in pure water and when using a mixture of water and ethylene glycol (EG) in a 1:1 ratio. Scale bar is 1 mm.

As discussed earlier, increasing the viscosity of the reactants effectively reduced the particle sizes during synthesis. However, this led to decreased reproducibility of the results as well. This effect can be explained by the instability in the contact area between the reactants, with difficulties emerging when aiming to control the laminar and turbulent flows affecting the stoichiometry of the synthesis reaction. To address this challenge, changing from diffusive mixing to turbulent mixing is proposed as a potential solution. An effective approach to introduce a turbulent flow is using an oil emulsion, where the reactants form microbubbles acting as microreactors with higher Reynolds numbers [31]. These microbubbles facilitate enhanced turbulent mixing, thereby promoting a more efficient contact and reaction kinetics between the reactants. This transition to turbulent mixing is promising for improving the reproducibility and control of particle synthesis processes. Figure 4 illustrates the internal structure of the chip designed to facilitate the formation of microbubbles—microreactors in an oil emulsion. Additionally, the images of the central channel are shown in Figure 4, demonstrating the path taken by the generated microbubbles, based on water and a mixture of water and ethylene glycol, as they flow through the chip towards the outlet.

**Figure 5:**
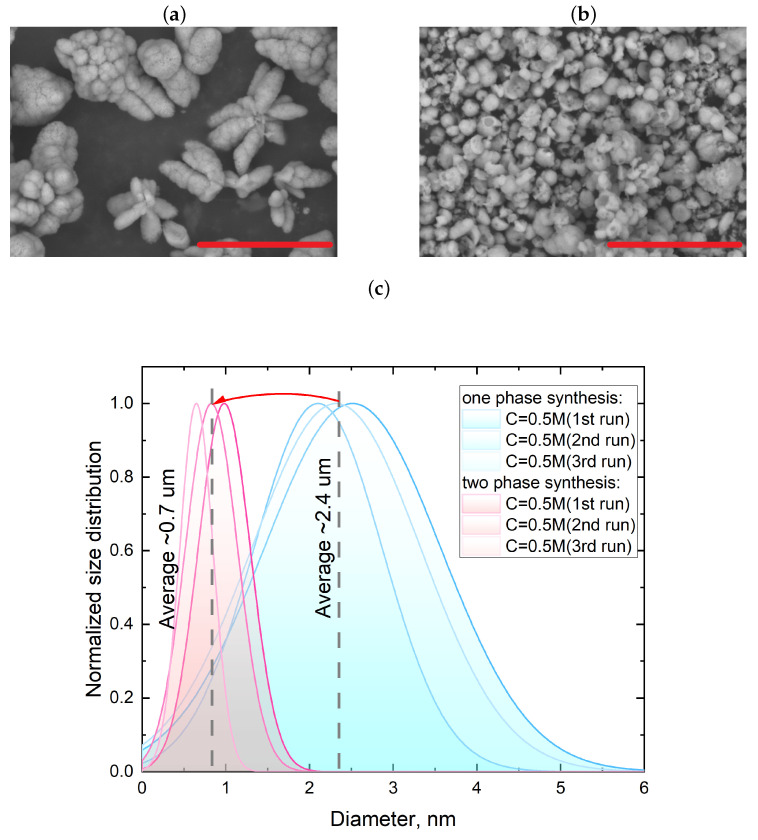
(**a**,**b**)—SEM images of calcium carbonate particles synthesized through one-phase and two-phase methods, respectively, using a reactant mixture with EG at a 1:1 ratio. (**c**)—Comparison of size distribution and average diameter of calcium carbonate particles synthesized in three consecutive runs using one-phase (blue curves) and two-phase (red curves) methods. Scale bar is 10 μm. Size distribution analysis performed using the methodology described in the Appendix A section on a data set provided by the SEM measurements.

As evident from the microphotographs presented in Figure 4, when using a mixture of water and ethylene glycol, the generated microbubbles exhibited a distinct elongated shape, unlike the microbubbles obtained with pure water. This behavior can be attributed to similar viscosities of castor oil and the water–ethylene glycol mixture [32]. Such a similarity in viscosities led to a modification in the microbubble formation regime. Notably, at the utilized oil flow rate of 0.01 mL/s and reactant flow rate of 0.005 mL/s, the formation of microreactors remained stable. As demonstrated in the previous section, increasing the viscosity of the reactant solution led to a reduction in the efficiency of calcium carbonate particle growth, resulting in a smaller average particle size and a narrower size distribution range. Additionally, it is shown that one-phase synthesis using reactant solutions with a higher viscosity can lead to the destabilization of the crystal growth process. Figure 5 presents the SEM images of the particles obtained through one-phase and two-phase synthesis with a concentration of 0.5 M in a water and ethylene glycol mixture in a 1:1 ratio.

**Figure 6:**
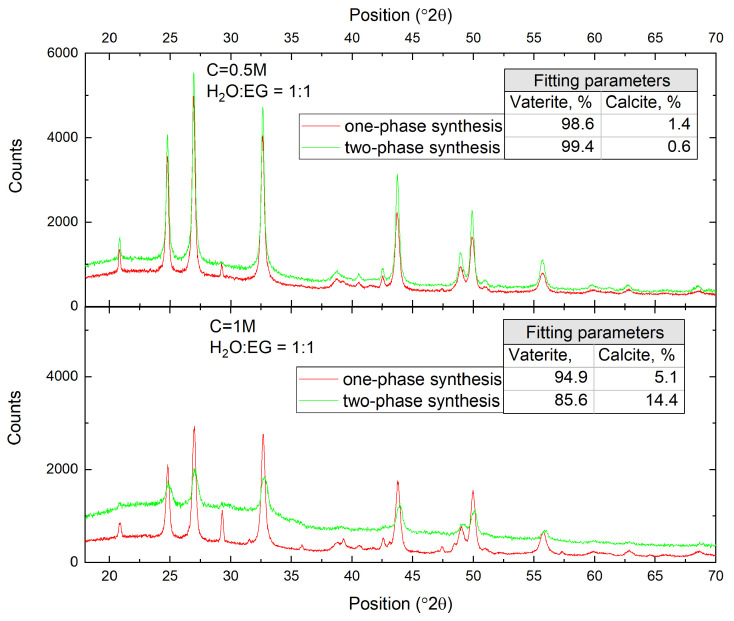
X-ray powder diffraction patterns of CaCO_3_ synthesized via one- or two-phase routes (red and green curves, respectively), at a reagent concentration of 0.5 M (top panel) and 1M (bottom panel) and water–EG ratio of 1:1. Inset: calcite–vaterite ratio calculated through full-profile analysis usng the Rietveld method.

As shown in Figure 5, transitioning from one-phase synthesis to two-phase synthesis resulted in a significant reduction in average particle diameter by more than three-fold, forming an ensemble of submicron-sized particles. However, a relatively low reproducibility still existed in particle sizes among the repeated syntheses. Noteworthy, in both cases, according to the XRD analysis of the synthesized particles, their polymorph composition was predominantly represented by the vaterite phase (Figure 6).

The characteristic peaks in the diffraction patterns at the angles 2θ equal to 20.77${}^{\circ }$, 24.70${}^{\circ }$, 26.90${}^{\circ }$, 32.60${}^{\circ }$, 38.65${}^{\circ }$, 43.65${}^{\circ }$, 48.75${}^{\circ }$, 49.70${}^{\circ }$, and 55.50${}^{\circ }$, corresponded to the vaterite crystallographic planes (004), (110), (112), (114), (211), (300), (304), (118), and (224). The peaks at the angles 2θ equal to 23.03${}^{\circ }$, 29.34${}^{\circ }$, 32.16${}^{\circ }$, 35.98${}^{\circ }$, 39.38${}^{\circ }$, 43.11${}^{\circ }$, 47.51${}^{\circ }$, 48.52${}^{\circ }$, and 57.30${}^{\circ }$ corresponded to the calcite crystallographic planes (012), (104), (006), (110), (113), (103), (202), (016), (018), and (122), respectively. All of the peaks found in the synthesized CaCO_3_ samples corresponded to two vaterite and calcite phases; a full-profile analysis using the Rietveld method confirmed that the dominant phase in the particles synthesized with a low reagent concentration was vaterite (∼99%), with a minor calcite inclusion (∼1%), while for the increased reagent concentration, calcite phase inclusion grew to ∼15%. This could be explained due to changes in pH level inside of the growing region of CaCO_3_ particles and the subsequent recrystallization of vaterite particles.

**Figure 7:**
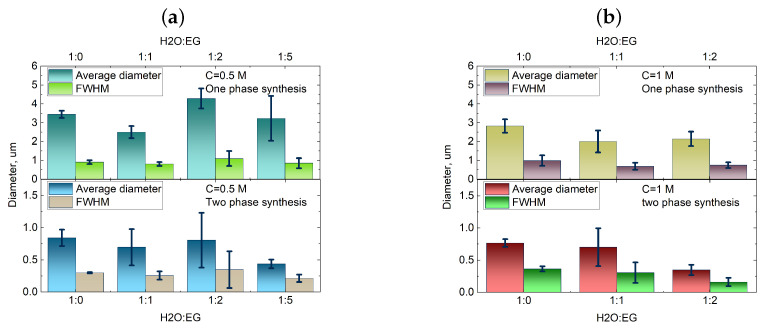
The dependence of the average diameter and size distribution (FWHM) of calcium carbonate particles on the synthesis type (top panel for one-phase synthesis and bottom panel for two-phase synthesis) and the water–ethylene glycol ratio in the reactant mixture and different reagent concentration ((**a**) 0.5 M, (**b**) 1 M). Average size and size distribution analysis performed using the methodology described in the Appendix A section on data set provided by the LSM measurements.

In Figure 7, the dependencies of the size distribution and average size of calcium carbonate particles are presented as a function of the synthesis type (one-phase or two-phase) and the viscosity of the reactant solution, controlled by varying the water–ethylene glycol ratio. These dependencies were collected as the average values across five iterations of each synthesis in order to present statistically sound data.

**Figure 8:**
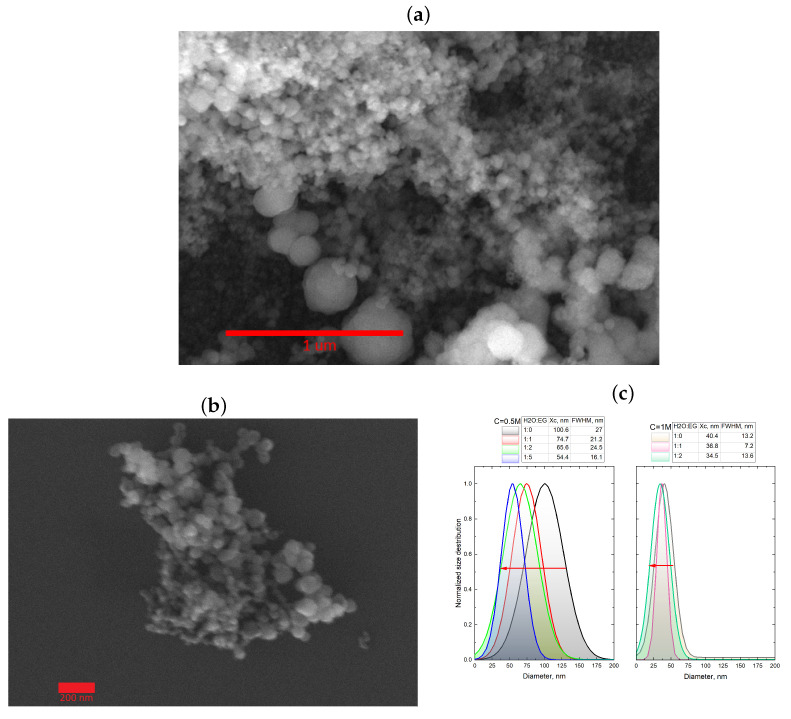
(**a**) SEM images of CaCO_3_ nanoparticles synthesized by the two-phase route in a water–EG ratio of 1:1 and concentration of 0.5 M (**a**) and 1 M (**b**). (**c**) Dependence of the average size and size distribution of calcium carbonate particles on the H_20_:EG ratio. Red arrows highlight the reduction in particles sizes during transition from low to high H_20_:EG ratio. Size distribution analysis performed using the methodology described in the Appendix A section on the data set provided by the SEM measurements.

As shown in Figure 7, transitioning from one-phase to two-phase synthesis resulted in a significant reduction in particle size by approximately 3–5 times across all of the cases. Moreover, there was a clear trend towards decreasing the average particle size with an increase in the viscosity of the reactant mixture. However, for the 0.5 M concentration, the effect of viscosity variation had minimal impact on the average particle size, but it led to reduced synthesis reproducibility. The results indicate that increasing the viscosity of the reactant solutions, achieved through the addition of ethylene glycol, led to a significant reduction in particle size. The two-phase synthesis approach, employing an emulsion of water and ethylene glycol as the solvent, resulted in the formation of submicron-sized particles, while the one-phase synthesis led to 3–4 μm particle sizes. Considering that a substantial quantity of submicron particles was produced using the two-phase synthesis method, their size approached the diffractive resolution limit of the confocal microscope (∼200 nm). To facilitate a more comprehensive analysis, the samples of particles synthesized using the two-phase microfluidic method were investigated using the small-angle scattering technique and scanning electron microscopy (SEM). Figure 8 depicts the SEM images obtained for the nanoscale CaCO_3_ particles synthesized by the two-phase route with various H_20_:EG ratios.

**Figure 9:**
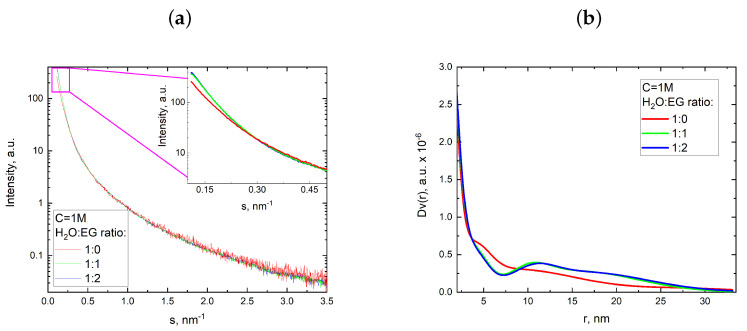
Experimental SAXS curves (**a**) and functions of particle volume distribution by size Dv(r) in the approximation of spherical particles (**b**) for CaCO_3_ synthesized with a reagent concentration of 1 M and various water–EG ratios.

As can be seen in Figure 8, the samples synthesized by the two-phase route contained a large amount of nanoparticles. It should be noted that this amount of nanometric CaCO_3_ particles was at least a few orders of magnitude greater than the number of micron-size particles (Figure 8a). The nanometric particles appeared only in samples provided by the two-phase synthesis method. A more comprehensive analysis of the average particle size and their size distribution revealed a positive trend towards reducing the size of the nanoscale particles from approximately 90 nm to around 30 nm, which correlated well with the gradual increase in viscosity of the precursor solutions (Figure 8c). As seen from the SEM images, the product of the two-phase synthesis from 1 M salt solutions were nanoparticles, which were suitable for analysis using small angle X-ray scattering (SAXS). SAXS can be very powerful to study the formation and growth of CaCO_3_ crystals, including their precipitation kinetics and the formation of amorphous calcium carbonate via in situ SAXS using synchrotron radiation source [33,34,35,36]. A paper from 2023 showed that SAXS in a flow system allowed for monitoring the temporal evolution of the particle radii and the impact of the concentration of precursors; this confirmed previous assumptions that precursors play a key role in determining the size of the stable particles obtained [33]. Unlike SEM, SAXS data represent the average of a very large number of colloidal particles, as all particles present in the illuminated volume contribute to scattering. Experimental SAXS curves and volume distribution functions for CaCO_3_ samples precipitated in the two-phase synthesis are presented in Figure 9.

The initial part of the scattering curves at s ≤ 0.5 nm${}^{-1}$ for the samples precipitated in a medium containing 50 and 70% of EG (the green and blue curves in Figure 9a) had sharper slopes than for the sample precipitated in pure water solutions (the red curve in Figure 9a). These sharper slopes corresponded to the presence of a larger proportion of large particles in the size range accessible to the experimental method, which is also reflected in Figure 9b. The functions of volume distribution of particles by size Dv(r) (Figure 9b) in the approximation of spherical particles, where r is the sphere radius, were calculated by the MIXTURE program (for details see materials and methods Section 2.4). For the three studied samples, the distribution was relatively wide and varied from 3 to 32 nm. To adequately characterize the particles in this size range, electron microscopy methods are not suitable as they limit the number of detected particles and provide no integral picture.

## 4. Discussion

The predominance of homogeneous compared with heterogeneous nucleation under the conditions of a microfluidic chip and highly supersaturated reaction mixture launches the mechanism leading to the stabilization of particles in the nanoscale and to a decrease in the width of the peak in their size distribution. The solubility of calcium carbonate in water–EG solutions decreased with the increased EG content, which reduced both the nucleation rate and the crystal growth rate [37].

As in the case of precipitation in large-volume tanks, at the microfluidic conditions, an increase in co-solvent content entailed a decrease in particle size, as did the use of more concentrated salt solutions [38,39,40]. The decrease in CaCO_3_ nanoparticle size after transition to a more concentrated reagent solution at a constant EG content can be attributed to the increased rate of nucleation. As the rate of nucleation increased and the critical nucleus size decreased, the process shifted towards the predominance of stable nucleus formation over their subsequent growth. Following Ostwald’s rule of selection, this indicated that as supersaturation increased, the rate of viable nucleus formation per unit time also escalated. Consequently, the ions from the solution were predominantly consumed when generating nucleation centers.

Commonly, when precipitating in large-volume tanks, the presence of an EG precursor stabilizes the vaterite polymorph [38,41].

In addition to better control over the crystallization process, crystallization in microfluidic conditions allowed for synthesizing vaterite particles of significantly smaller sizes compared with synthesized in large-volume tanks. The smallest CaCO_3_ spheres in large-volume tanks were obtained at the highest EG concentration (water–EG ratio of 1:5) and their size was 350–500 nm [39]. Here, we report significantly smaller vaterite particles that formed in microdroplets where the EG content varied from 50 to 75%. The particle diameters estimated from SAXS data were in the range of 6–64 nm, and the particle diameters calculated from the SEM images were in the range of 25–60 nm.

We believe that synchrotron SAXS, including a time-resolved version, is extremely promising for studying the in situ formation and growth of CaCO_3_ from supersaturated salt solutions under microfluidic conditions. By using this single experimental method, comprehensive data about the size, shape, mass density, number density, and aggregation behavior of the formed CaCO_3_ particles could potentially be obtained as a function of the reaction time. Detection of SAXS data in a flow system during synthesis could allow for a rapid analysis of the precipitate and variation in synthesis conditions, depending on the desired output characteristics.

Throughout this study, we engaged in multiple iterations of CaCO_3_ synthesis methods, leading to a gradual reduction in both a diameter and size distribution of the produced particles by two orders of magnitude. Table 1 summarizes the methods used in this study and the results yielded. We can conclude that microfluidics certainly provides a reliable means for the production of nanoscale CaCO_3_ particles, which was not possible even whenusing the best optimized conditions of the mass crystallization in the bulk.

The devised methodologies facilitate the production of suspensions containing nano- and submicron-sized vaterite particles from microfluidic chips, achieving concentrations in the range of 10–15 mg/mL. This process leverages 3D-printed microfluidic chips, as exemplified in Figure 1 and Figure 4. The scalability of the synthesis technology, coupled with the continuous generation of the reaction product at the prescribed flow rates within the microfluidic chips enables the synthesis of nanoparticles at a rate of up to 500 mg per hour. This achievement represents an order of magnitude increase in output compared with traditional bulk crystallization conducted in standard laboratory flasks and conventional reactors. Consequently, this advancement broadens the potential applications of nano- and submicron-sized vaterite particles.

## 5. Conclusions

In this study, we explored the complexity of calcium carbonate synthesis through a microfluidic approach. Indeed, the application of 3D-printed microfluidic chips in our experiments revealed their suitability for precise and controlled particle synthesis. The pursuit of smaller particle sizes through the adoption of the two-phase synthesis strategy yielded promising outcomes, showcasing the remarkable versatility of this approach. However, this advancement did come with a distinct trade-off in terms of synthesis reproducibility, shedding light on a critical aspect that demands further consideration.

We revealed a delicate balance between particle size reduction and synthesis reproducibility. While the transition to two-phase synthesis facilitated the generation of submicron particles ranging from 0.7 to 0.9 μm, achieving consistent particle sizes across synthesis attempts posed challenges due to increased reactant viscosity. This highlights the need for careful consideration of viscosity-related effects on particle formation dynamics.

Furthermore, our analysis ventured into the nanoscale, delving into the characterization of nanosized calcium carbonate particles using scanning electron microscopy. The obtained SEM images provided visual evidence of particles that remained beyond the spatial resolution limits of confocal microscopy. The detailed examination of these particles showcased a promising trend towards diminishing the particle size down to 30–60 nm, as the viscosity and precursor concentration were augmented. It is important to highlight that the synthesis route employed in this study led to the predominant formation of submicron CaCO_3_ particles in vaterite form (86–99%).

We have demonstrated that the continuous synthesis of nanosized calcium carbonate particles with a dominant vaterite polymorph offers an exciting pathway to finely adjust the properties of these particles for specific applications, underscoring the potential of this synthesis method. This study not only advances our understanding of microfluidic-based calcium carbonate synthesis, but also underscores the intricate interplay of parameters governing particle dimensions and synthesis reproducibility. Our findings shed light on the complexity of controlling the particle size while maintaining process stability, providing a foundation for further studies aimed at optimizing microfluidic synthesis strategies. Ultimately, the insights gained from this research contribute to the broader goal of tailoring particle characteristics for diverse applications, spanning from materials science to pharmaceuticals, and beyond.

## Figures and Tables

**Table 1:** Summary of CaCO_3_ synthesis methods used in this study.

Synthesis Type	Parameters	Results
One-phase with use of pristine reagent mixture	Concentration of 0.1, 0.33, 0.5, and 1 M	Diameter ∼ 3 μm, FWHM ∼ 1 μm
One-phase with use of EG and H_2_O reagent mixture	Concentration of 0.5 and 1 M	Diameter ∼ 2 μm, FWHM ∼ 0.7 μm
Two-phase with use of pristine reagent mixture reagent mixture	Concentration of 0.5 and 1 M	Diameter ∼ 0.7 μm, FWHM ∼ 0.3 μm
Two-phase with use of EG and H_2_O reagent mixture	Concentration of 0.5 and 1 M	Diameter ∼ 0.05 μm, FWHM ∼ 0.01 μm

**Figure A2:**
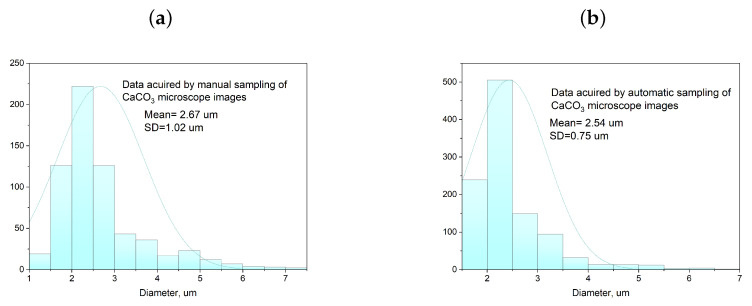
Size distributions of CaCO_3_ particles identified manually (**a**) and through digital image processing (**b**). Size distribution analysis performed on data set provided by LSM measurements. Mean and standard deviation were calculated through Gaussian fitting of experimental data.

## Data Availability

Data are available upon request.

## Abbreviations

The following abbreviations are used in this manuscript:

SAXS	Small-angle X-ray scattering
MSLA	Masked Stereolithography Apparatus
DLS	Dynamic light scattering
XRD	X-ray diffraction analysis

## Appendix A

In order to streamline the process of collecting statistical data on particle size distribution, a digital image processing procedure was developed. This procedure involves two primary stages: first, the application of the Canny edge detection operator to identify object boundaries in the image [42]. Second, the detection of circles along the edges using the Hough circle transform to determine the position and radius of recognized circles [43]. Figure A1 illustrates calcium carbonate microspheres captured using confocal microscopy and subjected to sequential Canny and Hough transformations (Figure A1c,d). It is worth noting that confocal microscopy images of objects with significant height non-uniformity may contain noise and elements outside the focal plane, rendering them unsuitable for digital processing. To address this issue, a Z-stack of microsphere images was recorded, capturing the focal plane at different positions along the Z axis (Figure A1a). Subsequently, the Z-stack was projected onto a Z-axis plane, and the pixel values were averaged based on the minimum value (Figure A1b). This technique effectively obtained images where all objects were in focus, eliminating distortions and noise.

As illustrated in Figure A1, the Canny and Hough transformations were proven to be effective at detecting circles in images. Moreover, this procedure exhibited a high level of reproducibility, given the limited number of variables employed in the transformations. Figure A2 presents histograms depicting the size distribution of microspheres obtained through both manual and digital processing of Figure A1a.

As depicted in Figure A2, both plots exhibited uniform size distributions. Notably, the histogram generated through digital image processing (Figure A2b) encompassed a significantly larger data set. Hence, it can be inferred that the chosen data processing algorithm was proven to be a reliable and efficient method for the initial analysis of spherical calcium carbonate particles. Additionally, it is worth mentioning that this algorithm is applicable to images acquired through electron microscopy.
